# Regulation on the metabolic synaptic pathway in the dorsal raphe nucleus in a rat model chronic olanzapine-induced body weight gain

**DOI:** 10.3389/fphar.2026.1942856

**Published:** 2026-09-23

**Authors:** Ping Huang, Jing Zhu, Zhimin Xia, Hua Li, Yanzhao Wang, Ke Zou, Tingfang Zhang

**Affiliations:** 1 Department of Endocrinology, The Fifth People’s Hospital of Jiujiang City, Jiujiang, Jiangxi Province, China; 2 Experimental Center, The School of Basic Medicine, Jiujiang University, Jiujiang, Jiangxi Province, China; 3 Affiliated Hospital of Jiujiang University, Jiujiang, Jiangxi Province, China

**Keywords:** body weight gain, dorsal raphe nucleus, olanzapine, proteomics, serotonergic synaptic pathway

## Abstract

**Objective:**

To explore the proteomic difference and mechanism in the dorsal raphe nucleus (DRN) of rats with body weight gain induced by long-term olanzapine administration.

**Methods:**

Thirty-six female Sprague-Dawley rats were randomly divided into two groups: an olanzapine treatment group (2.4 mg/(kg·d) and a vehicle control group (0.5% sodium carboxymethyl cellulose). Both treatments were administrated via drinking water for 24 continuous weeks. On the last day of 24th week, DRN samples were collected for proteomic analysis, quantitative real-time reverse transcription polymerase chain reaction (qPCR), Western blot (WB) analysis and bioassays. Differentially expressed proteins related to olanzapine treatment were subjected to bioinformatics analysis. Target genes were selected based on proteomic data for validation using qPCR and WB. Oxidative stress and inflammatory reaction were performed.

**Results:**

Body weight gain in the olanzapine group was significantly higher than in the control group starting from the week 18, exceeding 10% by week 24. Proteomic analysis identified 156 upregulated and 37 downregulated proteins in the DRN of the olanzapine-treated rats compared to the controls. KEGG analysis enriched these differentially expressed proteins into serotonergic, glutamatergic, and GABAergic synapses, alongside calcium signaling pathways related to olanzapine treatment. Protein-protein interaction (PPI) network analysis indicated that several olanzapine-associated differentially expressed proteins, including Ddc, Gchfr, Chrnb2, Tph2, Gad1, Grm1, Slc17a7, Gabra2, Gabra4, Gng3, Gng13, and Gnb4, were closely correlated to metabolic disorders. qPCR and WB confirmed that Tph2, the rate-limiting enzyme for 5-HT biosynthesis were significantly downregulated in the DRN of the olanzapine-treated rats (*p* < 0.01) compared to the controls; Furthermore, both the mRNA and protein expression levels of key genes within the glutamatergic synaptic (*Grm1, Slc17a7*), the GABAergic (*Gabbr2, Gng3*), and the calcium signaling (*Ryr3, ITPR1*) pathways were significantly upregulated (*p* < 0.05). Elevated levels of reactive oxygen species (ROS), malondialdehyde (MDA), and interleukin-1 beta (IL-1β) in the DRN indicate that chronic olanzapine treatment induces oxidative stress and an inflammatory reaction.

**Conclusion:**

Long-term olanzapine treatment induces significant body weight gain in female rats. This metabolic dysregulation is associated with alterations in multiple central pathways within the DRN, including the 5-HT synaptic signaling pathway.

## 1 Introduction

Olanzapine is a second-generation (atypical) antipsychotic widely used in the treatment of schizophrenia, bipolar disorder, and other psychotic conditions (Miyamoto et al., 2012). Its therapeutic efficacy is attributed to block postsynaptic dopamine D2 receptors to alleviate positive symptoms, and serotonin 5-HT2A receptor antagonism, which contribute to the improvement of negative symptoms (e.g., social withdrawal, apathy (Meltzer and Massey, 2011). Although olanzapine lowers the risk of extrapyramidal side effects commonly associated with first-generation antipsychotics, it is associated with significant metabolic adverse effects, including weight gain, dyslipidemia, and insulin resistance. After 6 weeks of olanzapine treatment, body weights have increased more than 7% from baseline in 77% of patients (Alonso-Pedrero et al., 2019). Six months of treatment in adolescent patients led to abnormal elevations in cholesterol, triglyceride, and fasting blood glucose levels (Arango et al., 2014; Smith et al., 2008; Gómez-Revuelta et al., 2020; Ma et al., 2024), which increases the risk of type II diabetes, cardiovascular and cerebrovascular complications. Mechanism studies of the metabolic side effects involved alteration in reward-related feeding behavior driven by the dopamine *D2* receptor antagonism, and the blockade of *H1* receptors and serotonin 5-HT2C receptor in the hypothalamic nuclei, which reduces satiety signaling, increases sedation, and promotes hyperphagia via hypothalamic-pituitary axis. The primary pharmacological intervention, metformin, produces only modest weight loss effects (Marteene et al., 2019). 5-HT receptor agonists show good efficacy in reducing weight but are limited in clinical use due to severe side effects (Escartín Pérez et al., 2021; Bazan and Fares, 2016), suggesting the existence of key targets within the central 5-HT system for regulating weight gain.

The dorsal raphe nucleus (DRN) is the principal center for central serotonin (5-HT) synthesis (Conde et al., 2023). The 5-HT neurons in the DRN project extensively to multiple hypothalamic nuclei. Through the release of serotonin, the DRN can precisely regulate the neuronal activity of various hypothalamic nuclei (Pereira et al., 2024; Liu et al., 2024; Peng et al., 2025). The DRN, as the source of central 5-HT, has received far less attention. In the present study, we utilized liquid chromatography-tandem mass spectrometry (LC-MS/MS) to profile differentially expressed proteins in the DRN of a chronic olanzapine-induced weight gain rat model. To elucidate the molecular mechanisms underlying these olanzapine-driven metabolic dysfunction, we further validated potential downstream targets and pathways by assess their respective levels of mRNA and proteins.

## 2 Materials and methods

### 2.1 Ethical approval

The animal study was approved by the Ethics Committee for Animal Experimentation of the Fifth Hospital, Jiujiang, Jiangxi (approval No. 2021–2). All experiments were conducted during the daytime in accordance with the Regulations on the Administration of Laboratory Animals of the People’s Republic of China.

### 2.2 Animals

Healthy two-month-old Sprague Dawley (SD) female rats, weighing 170–210 g, were obtained from Hunan Silaike Jingda Laboratory Animal Co. (Changsha, PR. China). The rats were fed a standard diet with *ad libitum* access to food and water, housed under controlled conditions: a room temperature 22 °C–24 °C, 40%–60% humidity, a 12-h light/dark cycle, and adequate ventilation.

### 2.3 Experimental design and olanzapine treatment

To establish an olanzapine-induced weight gain rat model, 36 SD female rats (based on a power calculation of 96%) after a 2-week acclimatization period were divided into a control group (drinking water containing a 0.5% sodium carboxymethyl cellulose, CMC-Na solution) and an olanzapine treatment group (drinking water containing olanzapine) using a body weight-based serpentine sorting method. Olanzapine tablets (10 mg per tablet, Jiangsu Haosen Pharmaceutical Group Co., Suzhou, China) were used to prepare a fresh olanzapine solution by dissolving the tablets in a CMC-Na solution. The concentration of olanzapine (mg/L) in the drinking water was calculated daily using the following formula: [Total body weight (kg) of the olanzapine group on the day before preparation × 2.4 mg/kg] ÷ [Total drinking water volume (L) consumed by the olanzapine group in 24 h prior to preparation]. Initially, the olanzapine concentration was adjusted daily based on body weight and total drinking water consumption. Once the daily water intake per rat stabilized at approximately 20 mL, the concentration was adjusted weekly. After 24 weeks of treatment, the modeling was considered successful if the average body weight of the olanzapine group exceeded that of the control group by more than 10%. Body weight measurements were taken daily during the initial period for concentration adjustment and once per week throughout the remaining experiment.

### 2.4 DRN tissue preparation

On the last day of 24-week treatment, the rats were deeply anesthetized and perfused transcardially with cold PBS followed by a 4% paraformaldehyde solution. Subsequently, each DRN was carefully isolated in the sagittal plane, then placed into a 1.5 mL Eppendorf tube and quickly frozen in liquid nitrogen for storage.

### 2.5 Mass spectrometry (MS) analysis

Six DRN tissue samples from the two groups were randomly selected for analysis. Samples underwent lysis (1.5% SDS/100 mM Tris-HCl, pH = 8.5), tissue homogenization, protein denaturation, acetone precipitation, centrifugation, and pellet dissolution (8 M Urea/100 mM Tris-HCl, pH = 8.5) to obtain protein complexes. This was followed by trypsin digestion, iTRAQ labeling, reaction termination, centrifugation at 12,000 *g* to collect supernatant, desalting with SDB columns, drying, and storage at −20 °C.

Mass spectrometry analysis was performed using an UltiMate 3000 RSLCnano nano-flow liquid chromatography system (Thermo, Waltham, MA, USA) coupled with a timsTOF Pro mass spectrometer (Bruker, Karlsruhe, Germany). Twelve peptide samples were sequentially loaded via an autosampler, bound to a C18 trap column, and then eluted onto an analytical column (75 μm × 15 cm, 1.7 µm particle size, 100 Å pore size, IonOpticks) for separation. Data were acquired in diaPASEF mode. Capillary voltage was set to 1500 V. The scan range for both MS1 and MS2 spectra was set to 100–1700 m/z. The ion mobility range was set to 0.6–1.6 Vs/cm^2^. Accumulation time and ramp time were set to 50 m. The diaPASEF acquisition windows were configured using timsControl software based on the m/z-ion mobility distribution pattern. Collision energy was linearly decreased from 59 eV at 1/K0 = 1.6 Vs/cm^2^ to 20 eV at 1/K0 = 0.6 Vs/cm^2^ based on ion mobility. Each sample was analyzed twice (technical replicates). Data with a coefficient of variation (CV) < 15% were included in the analysis to ensure quantitative reliability.

Mass spectrometry data were analyzed using the library-free mode of DIA-NN software (version 1.9.1). The UniProt rat proteome reference database was used for searching. Key search parameters were as follows: Precursor ion generation enabled for theoretical spectral library prediction; Trypsin/P with up to 2 missed cleavages allowed; Carbamidomethyl (C) set as fixed modification; Oxidation (M) and Acetylation (protein N-terminal) set as variable modifications; MS1 and MS2 mass tolerances set to 15 ppm; Match-between-runs (MBR) enabled; Heuristic protein inference enabled; False discovery rate (FDR) set to 1%. Proteomic differential analysis used a two-sided *t*-test, with proteins showing a Benjamini–Hochberg adjusted *p* value < 0.05 considered significantly differentially expressed; a fold change threshold of >1.2 or <0.833 was set. Functional annotation of identified proteins was extracted from GO, KEGG, and COG databases. After obtaining annotation information for all proteins identified by MS, information related to the differentially expressed proteins was extracted. Categories and counts were summarized, and functional enrichment analysis was performed using a hypergeometric test. Annotation terms with *p* < 0.05 and containing ≥3 differentially expressed proteins were screened as significantly enriched functional categories relevant to the experiment.

### 2.6 Quantitative real-time PCR (qPCR)

Three DNR samples from the two groups were used for total RNA extraction using Trizol reagent. cDNA was synthesized using an RT First Strand cDNA Synthesis Kit (Servicebio Technology Co., Wuhan, China) following the manufacturer’s instructions for reverse transcription. Reaction conditions in a PCR thermal cycler: 25 °C for 5 min, 42 °C for 30 min, 85 °C for 5 s qPCR primer sequences are listed in Table 1, synthesized by Service bio. qPCR was performed using SYBR Green reagent kits on a Bio-Rad real-time PCR system. Reaction conditions: 95 °C for 10 min; 40 cycles of 95 °C for 15 s, 60 °C for 30 s; followed by a melt curve from 65 °C to 95 °C. Quantification was conducted using the comparative ΔCt method, with the mean expression level of *GAPDH* used for normalization.

**TABLE 1:** Sequences of qRT-PCR primers.

Primers	Sequence (5′-3′)
*Tph2*-F *Tph2*-R	AAGGTCAACTGCGGGCGTA TCTTGGAAGGTGGTGATTAGGC
*Cacna1c* -F *Cacna1c* -R	TATGCTACCAGAATGGCGGAG GGTGTTGTAGTAGGAGGCGATGT
*Prkcb* -F *Prkcb* -R	TGCTGTCACTTCAGGATGAAAGG TGCTGCCTTGGACACCTTTATC
*Prkcg* -F *Prkcg* -R	GTCAAGCGTTACAACTGGACCTAT GCAGAAGCAGACCACAACCCT
*Camk2a* -F *Camk2a* -R	GGGGTACATTGTCACTCAGATTCCT ATAGTGAACACGGGCTGCTGAA
*Map3k5*-F *Map3k5*-R	ATGGAAGGTTACATCGGAGTGG TGACCCAGATCCCATCGTAGG
*Ryr3*-F *Ryr3*-R	GACGACGACAAGAAGAAGAC TCCAGCAGCAGTAGTAGTGT
*Itpr1*-F *Itpr1*-R	ATGGACGACGACAAGAAGAC TCAGCAGCAGTAGTAGTGA
*Mtor*-F *Mtor*-R	AGTGGGCTCCTGTGAATGAC GCTGAGGGTCTTCACACACA
*GAPDH*-F *GAPDH*-R	CTGGAGAAACCTGCCAAGTATG GGTGGAAGAATGGGAGTTGCT

### 2.7 Western blot analysis

DRN samples were lysed, followed by separation via SDS-PAGE and transferred onto PVDF membranes (Millipore, Waltham, MA). Blotting reaction was as routinely performed. The primary antibodies, anti- Cacna1c, Itpr1, Tph2, β-actin polyclonal antibodies were purchased from Proteintech (Rosemont, IL); anti-Mtor polyclonal antibody were from abcam (Cambridge, UK); anti-Map3k5 polyclonal antibody were from Abclonal (Wuhan, P.R.China); anti-Prkcb and Ryr3 were from Thermo (Waltham, MA); and anti-rabbit fluorescent-labelled secondary antibodies were purchased from Cell Signaling Technology (Danvers, MA). The primary antibodies were used with 1:500–2000 dilution and the second antibodies were used with a 1:500 solution. An ECL chemiluminescent ultrasensitive detection kit was purchased from Yeasen, Shanghai, China. Protein imaging was taken using the Chemiluminescence imaging system (Tianneng, Shanghai, China). Analysis and quantification of immunoblot images were performed using ImageJ software (NIH, Bethesda).

### 2.8 Reactive oxygen species (ROS) detection

The DRN cellular ROS concentration was measured using DCFDA - ROS Assay Kit (Biyuntian, Shanghai, P.R. China) according to the manufacturer’s protocol. 300–400 μm thick DRN slices were prepared with a vibratome and slices were allowed to recover in oxygenated (95% O_2_/5% CO_2_) aCSF at 30 °C–32 °C for at least 60 min before staining. ROS analysis kit was purchased from Biyuntian. Transfer slices to a 24-well plate containing the DCFH-DA working solution (dilute the 10 mM stock solution 1:1000). Incubate at 37 °C for 45–60 min in the dark with gentle shaking, then transfer slices to new well containing pre-warmed PBS, wash 3 times. Tissue sections were subjected to autofluorescence quenching, washed with PBS, and nuclei were counterstained by incubating with DAPI in the dark for 10 min. Sections were mounted with anti-fade mounting medium. Images were observed under a fluorescence microscope and whole slides were scanned using a PANNORAMIC panoramic scanner. Images were viewed using CaseViewer 2.4 software, and fluorescence intensity was analyzed using Quant Center 2.3 software (DAPI excitation/emission wavelengths: 330–380/420 nm; FITC: 465–495/515–555 nm; CY3: 510–560/590 nm). Using the TMA plugin in Quant Center 2.3 analysis software, the tissue outline was defined, and the software automatically identified the tissue. Using the PatternQuant module in Quant Center 2.3, the red area (containing positive signal) was distinguished from the tissue area. The tissue part (Mask Area) was used as the tissue area. Within the red area hierarchy, HistoQuant was added to calculate the area and intensity of the positive part, and fluorescence intensity was calculated based on grayscale values.

### 2.9 Biochemical analysis

All the biaochemical analysis kits were purchased from Biyuntian. Detection of interleukin-1 beta (IL-1β) by ELISA. DRN samples were homogenized in pre-cooled buffer and centrifuged at 2000–3000 r/min for 20 min at 4 °C. The supernatants were collected for analysis. Samples and diluted standards were added to the pre-coated microtiter plate. The plate was gently shaken and incubated for 30 min at 37 °C. After repeated 3–5 times wash, HRP-conjugated antibody was added to each well and incubated for another 30 min at 37 °C. The washed wells were added freshly prepared TMB substrate solution and incubated in the dark for 10 min at 37 °C. Stop solution was added to each well to terminate the reaction and measurement was performed for OD at 450 nm using a microplate reader. A standard curve was constructed by plotting the mean OD value of each standard against its concentration. The concentrations of IL-1β in the samples were calculated by interpolating their OD values onto the standard curve. After washing, enzyme conjugate was added for a second incubation. TMB substrate was added for color development in the dark for 10 min, then stop solution was added. OD values were measured at 450 nm, and concentrations were calculated using the standard curve.

The malondialdehyde (MDA) kit was used to detect MDA level as the kit instruction. The ratio of tissue to extraction buffer was adjusted between 1:5 and 1:10. Reaction mixtures were incubated in a 90 °C–95 °C water bath for 30 min, cooled on ice, centrifuged, and absorbance was measured at 532 nm. The ΔA was calculated, and MDA content was determined.

### 2.10 Statistical analysis

Data were statistically analyzed using the SPSS 27.0 software. Measurement data are expressed as mean ± standard deviation (mean ± SD). We first tested the assumptions of normality and homoscedasticity for all data. For continuous variables with a normal distribution, data from qPCR, Western blot, ROS, and biochemical assays were analyzed using Student’s t-test. When variances were unequal, Welch’s *t*-test was used instead. Non-normally distributed quantitative data were analyzed using the Mann-Whitney U test. Body weight changes over time were assessed via repeated measures ANOVA with Sidak-adjusted *post hoc* tests. A *p*-value < 0.05 was considered statistically significant.

## 3 Results

### 3.1 Continuous drink of olanzapine-containing water for 24 weeks induced weight gain in female rats

One of the side effects of long-term administration of olanzapine leads to weight gain in patients. To investigate the underlying mechanisms, we established a chronic olanzapine-induced weight gain model in rats. Since female rats, but not male rats, exhibit significant weight gain after chronic olanzapine treatment according to previous literature (Davey et al., 2012), we used female rats in the present study. Successful weight gain is defined as an increase in body weight exceeding 10% compared to the control after olanzapine treatment. As illustrated in Figure 1 and Table 2, after 24 weeks of continuous olanzapine treatment, the differences in weight gain between the two groups became statistically significant from the 18th week onward. At week 24, the average weight in the olanzapine group was more than 10% higher than that in the control group. Thus, we successfully obtained chronic olanzapine-induced weight gain model in rats.

**FIGURE 1:**
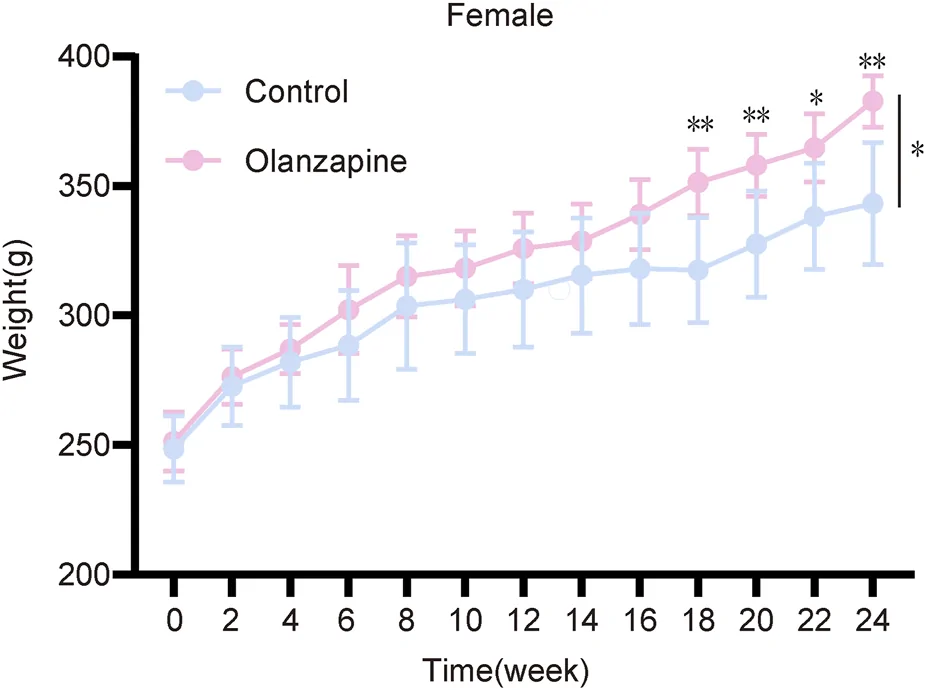
The weight of female mice. *p < 0.05, **p < 0.01.

**TABLE 2:** The weight of female mice (g) (Mean ± SD).

Sex	Group	N	Baseline	8th week	16th week	24th week
Female	Control	12	248.5 ± 12.83	303.7 ± 24.44	318.1 ± 21.56	343.3 ± 23.51
	Olanzapine	12	251.4 ± 11.26	315.1 ± 15.62	339.0 ± 13.60	382.7 ± 9.985

### 3.2 Chronic olanzapine treatment induced alterations related to energy metabolism

Next, we performed a proteomic analysis to compare protein expression profiles in DRN between the control and olanzapine-treated rats. Using the criteria of ratio >1.2 or ratio <0.833 and *p* < 0.05, a total of 193 differentially expressed proteins were identified in the olanzapine group compared to the control group, including 156 upregulated and 37 downregulated proteins, as shown in the Volcano plot (Figure 2A) and Heatmap (Figure 2B).

**FIGURE 2:**
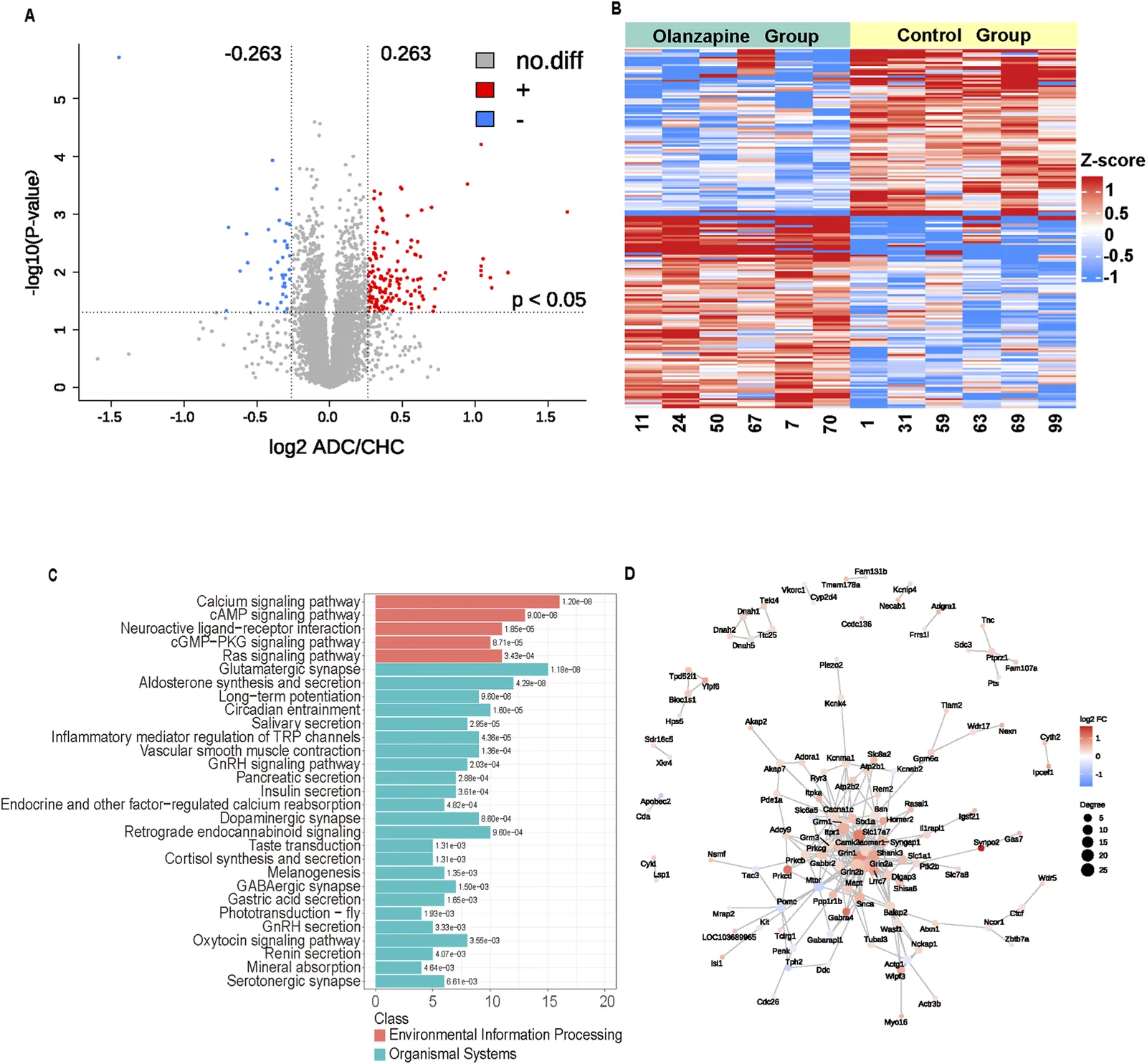
The proteomics of control and olanzapine group **(A)** Volcano plot shows 156 upregulated (red) and 37 downregulated (blue) proteins between the two group, with a threshold of |log_2_ADC/CHC| ≥ 0.263 and FDR <0.05. ADC: olanzapine group; CHC: control group **(B)** The heatmap shows the level of proteins between two group **(C)** KEGG pathway enrichment analysis of differentially expressed proteins between two groups **(D)** The PPI network among the differentially expressed proteins in the two groups.

Based on a review of relevant literature from databases such as PubMed, Wanfang Data, and CNKI, 20 differentially expressed proteins closely related to central nervous system signaling pathway regulation (14 upregulated and 6 downregulated) are listed in detail in Table 3.

**TABLE 3:** 20 differentially expressed proteins related to central nervous system signaling pathway.

UniProt ID	Gene	Protein	FoldChange	Padjust
A0A0A0MY31	*Itpr1*	Inositol 1,4,5-trisphosphate receptor type 1	1.5545	0.0261
A0A0G2JTL4	*Kit*	Mast/stem cell growth factor receptor	0.6144	0.0014
A0A0G2JX74	*Mtor*	Serine/threonine-protein kinase mTOR	0.6635	0.0098
A0A0G2JZK7	*Slc8a2*	Sodium/calcium exchanger 2	1.6466	0.0126
D3ZW27	*Map3k5*	Mitogen-activated protein kinase kinase kinase 5	1.5290	0.0098
D4A0U0	*Prkcd*	Protein kinase C	2.1504	0.0126
E9PT56	*Cacna1c*	Voltage-dependent L-type calcium channel subunit alpha-1C	1.5404	0.0483
F1LPJ2	*Ryr3*	Ryanodine receptor 3	1.5197	0.0101
F1LS36	*Prkcb*	Protein kinase C	1.5479	0.0020
G3V7U1	*Grm1*	Glutamate receptor, metabotropic 1, isoform CRA b	1.5795	0.0053
G3V746	*Grin2b*	Glutamate receptor ionotropic, NMDA 2B	1.5919	0.0162
P11275	*Camk2a*	Calcium/calmodulin-dependent protein kinase type II subunit alpha	1.6330	0.0341
P11505	*Atp2b1*	Plasma membrane calcium-transporting ATPase 1	1.5037	0.0229
P11506	*Atp2b2*	Plasma membrane calcium-transporting ATPase 2	1.5376	0.0318
P14173	*Ddc*	Aromatic-L-amino-acid decarboxylase	0.5685	0.0048
P17105	*Itpka*	Inositol-trisphosphate 3-kinase A	1.5018	0.0210
P63319	*Prkcg*	Protein kinase C gamma type	1.5835	0.0066
Q8CGU9	*Tph2*	Tryptophan 5-hydroxylase 2	0.6524	0.0097
Q8K422	*Pomc*	Pro-opiomelanocortin	0.6167	0.0340
Q0VGK0	*Gabarapl1*	Gamma-aminobutyric acid receptor-associated protein-like 1	0.6072	0.0157

KEGG pathway enrichment analysis of differentially expressed proteins between the two groups indicated that 35 pathways were enriched under the ‘Organismal Systems category’. The top 24 pathways with significant enrichment (p < 0.01) included glutamatergic, GABAergic, and serotonergic synapses (Figure 2C). Twelve pathways were enriched under ‘Environmental Information Processing’. The top pathway with the most significant enrichment (p < 0.001) was the Calcium signaling pathway (Figure 2C).

The PPI network among the differentially expressed proteins in the two groups was analyzed using the STRING database. The differentially expressed proteins Ddc, Tph2, Gad1, and Gchfr in the PPI network nodes are involved in neurotransmitter (including 5-HT) synthesis and metabolism; Grm1, Gabra2, Gabra4, Chrnb2, Gng3, Gng13, Gnb4, and Slc17a7 are involved in synaptic signal transmission (Figure 2D).

### 3.3 Validation of synaptic signaling pathway in DRN of olanzapine treated rats

Using a threshold of fold change ≥1.5 or ≤0.67 and *p* < 0.05, we selected seven proteins, which are associated with serotonergic, glutamatergic, and GABAergic synapses and act as hub nodes in the protein-protein interaction network, for validation using qPCR and Western blotting. As shown in Figure 3A, the mRNA expression level of the gene *Tph2* (p < 0.0001)*, Mtor* (p = 0.0006) in the serotonergic synaptic pathway were significantly downregulated in the olanzapine-treated group compared to the control group. The mRNA expression levels of genes involved in the glutamatergic synapse, GABAergic synapse, and calcium signaling pathway [*Cacna1c* (p = 0.0002)*, Map3k5* (p = 0.0003)*, Prkcb* (p = 0.0006)*, Ryr3* (p = 0.017)*, ITPR1* (p < 0.0001)] were upregulated in the drug-treated group compared to the control group (Figure 3A). In addition, the corresponsive protein levels [Ryr3 (p < 0.0001), Mtor (p = 0.005), Itpr1 (p = 0.0022), Cacna1c (p = 0.0002), Map3k5 (p = 0.0002), Prkcb (p = 0.0022), Tph2 (p < 0.0001)] were confirmed by Western blot as in Figures 3B,C.

**FIGURE 3:**
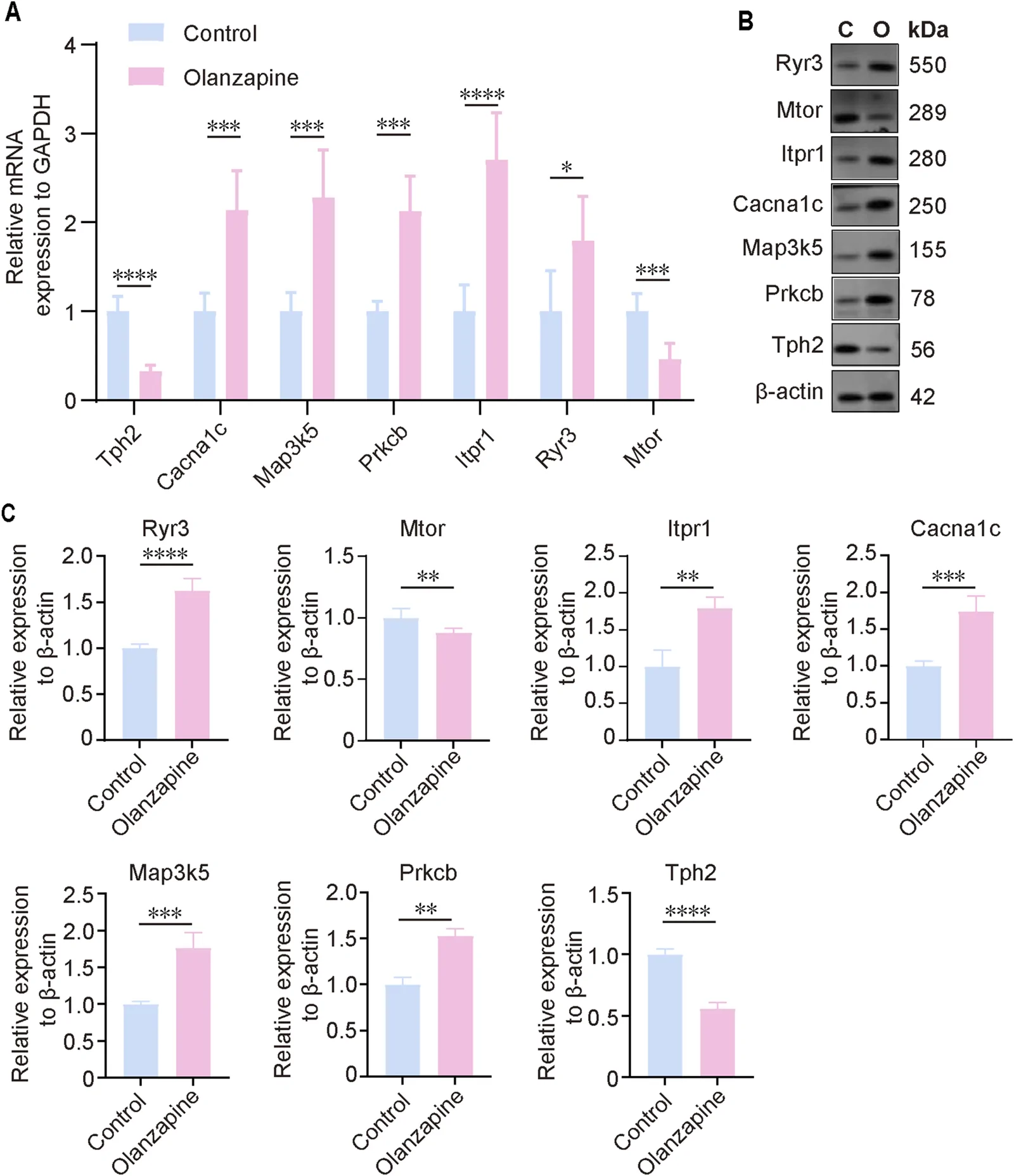
Confirmation of synaptic signaling pathway in DRN. **(A)** The mRNA expression levels of *Tph2*, *Cacna1c*, *Map3k5*, *Prkcb*, *Itpr1*, *Ryr3*, *Mtor*. **(B,C)** The protein expression levels of Tph2, Cacna1c, Map3k5, Prkcb, Itpr1, Ryr3, Mtor. **(C)** control group; O: olanzapine group. **p < 0.01, ***p < 0.001, ****p < 0.0001.

### 3.4 Chronic exposure to olanzapine induced neuroinflammation and oxidative stress in DRN

To evaluate the impact of olanzapine exposure, we measured levels of IL-1β, MDA, and ROS in both the control and treatment groups. Our analysis revealed that treatment with olanzapine induced a significant state of neuroinflammation and oxidative stress. Compared to the control group, the olanzapine-treated group exhibited a marked elevation in the pro-inflammatory cytokine IL-1β (*p* = 0.0022, Figure 4C). Concurrently, markers of oxidative damage were substantially increased, as evidenced by significantly higher levels of MDA (*p* = 0.03, Figure 4C), a key product of lipid peroxidation, and elevated ROS production (*p* = 0.0014, Figures 4A,B). These findings indicate that olanzapine exposure is associated with heightened inflammatory and oxidative stress responses in DRN.

**FIGURE 4:**
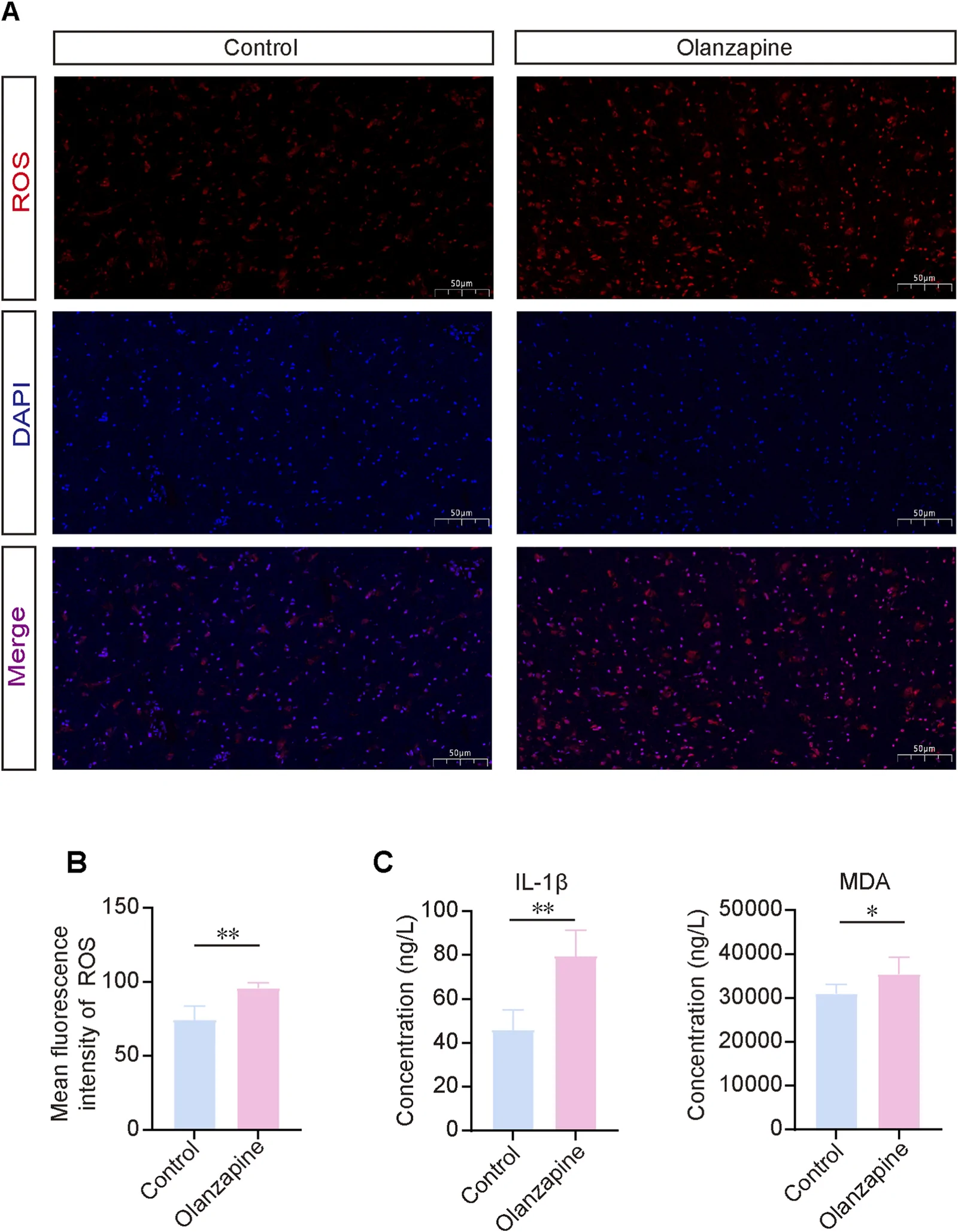
Chronic exposure to olanzapine leaded to neuroinflammation and oxidative stress in DRN. **(A,B)** The level of ROS between two groups. **(C)** The level of pro-inflammatory cytokine IL-1β and MDA. *p < 0.05, **p < 0.01.

## 4 Discussion

Approximately two-thirds of all central 5-HTergic neurons are located in the DRN. These neurons project extensively to widespread brain regions (Nectow et al., 2017), particularly to the pro-opiomelanocortin (POMC) centers of the hypothalamus, which express 5-HT2A/2C receptors and serve as key sensors for energetic homeostasis. While the mainstream hypothesis centers on the receptor-level blocking effects of olanzapine within the hypothalamus (Bickerdike, 2003; Fernø et al., 2011), existing animal models are largely limited to short-term interventions of 1–7 weeks. Consequently, it is unclear whether long-term clinical administration impacts the brain; specifically, previous research neglects to explore whether exposure of olanzapine directly injures serotonergic neurons in the DRN. Here, we analyzed the protein profile in DRN of the long-term olanzapine treated female rats. Our result showed that 24 weeks of olanzapine treatment induced over 10% more weight gain than controls, confirming the successful establishment of the model. Significantly, proteomic analysis of the chronically treated DRN identified 193 differentially expressed proteins (156 upregulated, 37 downregulated). Bioinformatics analysis indicated these proteins are associated with transporter activity, intercellular signal transduction, synaptic plasticity and synaptic signaling, specifically enriching into serotonergic, glutamatergic, GABAergic, and calcium signaling pathways. Additionally, seven significant altered candidate genes—*Cacna1, ITPR1, Map3k5, Mtor, Prkcb, Ryr3* and *Tph2* —were selected for validation. Both mRNA and protein levels of *Tph2* and *Mtor* were significantly downregulated, whereas other five candidate genes were significantly upregulated. Elevated ROS, MDA, and IL-1β levels indicate that chronic olanzapine treatment induces oxidative stress and inflammation in the DRN. Our proteomic profiling of the DRN aligns with the framework reviewed by Pagi (Pagi, 2026), linking altered protein expressions with the neuro-metabolic and neuroinflammatory mechanisms driving olanzapine-induced dysfunction. By contextualizing localized DRN findings within this broader model including serotonergic signaling and metabolic regulation, our study strengthens the translational potential for mitigating antipsychotic-associated weight gain.

Long-term exposure of olanzapine in DRN might lead to 5-HT biosynthesis and less release of 5-HT. Tph2 is predominantly found within 5-HT neurons of the DRN and is the rate-limiting enzyme for 5-HT biosynthesis. Central 5-HT has an appetite-suppressing effect (Goit et al., 2022). 5-HT receptors are divided into 7 families (5-HT1R to 5-HT7R), comprising 14 subtypes. Except for the 5-HT3 receptor, which is a ligand-gated ion channel, the others belong to the G protein-coupled receptor (GPCR) family (Vahid-Ansari and Albert, 2021). 5-HT binding to 5-HT1A/1B and 5-HT2A/2C receptors can regulate feeding behavior, thereby controlling body weight. Our proteomic analysis revealed that olanzapine significantly downregulated *Tph2* mRNA and protein expression in DRN of rats (*p* < 0.01), which may imply less synthesis of 5-HT in the neurons. In addition, mTORC1 is excessively upregulated, it can, via a potent feedback loop mediated by the insulin/insulin-like growth factor receptor (insulin/IGF receptor), inhibit Akt/mTORC1 pathway activity. Inhibition of mTORC1 activity can induce depletion of synaptic vesicles in monoaminergic presynaptic terminals, reducing the readily releasable pool (RRP) in 5-HT neurons of DRN and thereby lowering 5-HT levels (Sanz-Castillo et al., 2023; Garro-Martínez et al., 2021). Thus, downregulation of 5-HT biosynthesis due to less Tph2 expression and lower level 5-HT release by inhibition of mTORC1 will inhibit the 5-HTergic nervous system signaling pathway and promote feeding and body weight gain.

Several elements increased in the calcium signaling pathway and may play a role oxidative stress/inflammation in DRN, as indicated in Figure 4. The soluble second messenger inositol 1,4,5-trisphosphate (IP_3_), binds to and activates ITPR1 in the endoplasmic reticulum, together with RYR3 activation, releases Ca^2+^ into the cytoplasm. This may subsequently upregulate the CACNA1C, mediating increased extracellular Ca^2+^ influx (Chang and Liou, 2016; Ramírez et al., 2021), thereby modulating CAMK2A and the mTOR signaling pathway (Zhang et al., 2024; Qi et al., 2021). As intracellular Ca^2+^ and diacylglycerol (DAG) increase, they activate PRKCB, triggering downstream MAPK inflammatory cascade reactions (Song et al., 2022; Tao et al., 2022). Elevated cytosolic Ca^2+^ concentration resulting from PLC activation is a key factor for activating apoptosis signal-regulating kinase 1 (ASK1/Map3k5) (Patel et al., 2019). Map3k5 participates in neuronal cell inflammatory damage by stimulating c-Jun N-terminal kinase (JNK) and p38 MAPK signaling pathways. Inhibition of neuronal cell ASK1 activity can reduce the levels of pro-inflammatory factors Tnf-α and Il-1β (Meng et al., 2023), suggesting that ASK1 activation can induce neuronal inflammatory damage. Thus, long-term oxidative stress combined with chronic low-grade inflammatory response may lead to central 5-HT neuronal synaptic damage, ultimately downregulating Tph2 expression and reducing 5-HT biosynthesis and release.

In summary, chronic olanzapine administration is associated with the modulation of various signaling pathways within the DRN, specifically those governing serotonergic, glutamatergic, GABAergic, and calcium dynamics. While olanzapine is known to antagonize 5-HT_2_A/5-HT_2_C receptors, our findings suggest olanzapine treatment may also correlates with attenuated 5-HT signaling through indirect mechanisms, such as downregulating Tph2 expression and impairing 5-HT synthesis and release via localized oxidative stress and inflammation.

Several limitations of this study should be noted. First, to expedite the experimental timeline, only female rats were utilized; future investigations should incorporate both sexes to evaluate potential gender-dependent metabolic differences by increasing the dose or treatment duration. Second, tracking body weight alone provided limited insight into broader metabolic dysfunction. Future research should evaluate parameters such as food intake, body composition, insulin sensitivity, glucose tolerance, lipid profiles, and energy expenditure to enrich the biological analysis. Third, proteomic profiling was restricted to the DRN; expanding this analysis to other critical brain structures, such as the striatum and hypothalamus, would offer valuable comparative insights. Fourth, the validation phase was constrained by a relatively small sample size, which could be expanded in future studies to strengthen statistical power. Finally, the lack of functional validation experiments limits our ability to definitively link the altered molecular pathways to olanzapine-induced weight gain.

## Data Availability

The datasets presented in this study can be found in online repositories. The names of the repository/repositories and accession number(s) can be found in the article/supplementary material.

## References

[B1] Alonso-Pedrero L. Bes-Rastrollo M. Marti A. (2019). Effects of antidepressant and antipsychotic use on weight gain: a systematic review. Obes. Rev. 20, 1680–1690. 10.1111/obr.12934 31524318

[B2] Arango C. GiráLDEZ M. MercháN-Naranjo J. Baeza I. Castro-Fornieles J. Alda J. A. (2014). Second-generation antipsychotic use in children and adolescents: a six-month prospective cohort study in drug-naïve patients. J. Am. Acad. Child. Adolesc. Psychiatry 53, 1179–1190. 10.1016/j.jaac.2014.08.009 25440308

[B3] Bazan I. S. Fares W. H. (2016). Review of the ongoing story of appetite suppressants, serotonin pathway, and pulmonary vascular disease. Am. J. Cardiol. 117, 1691–1696. 10.1016/j.amjcard.2016.02.049 27018933

[B4] Bickerdike M. J. (2003). 5-HT2C receptor agonists as potential drugs for the treatment of obesity. Curr. Top. Med. Chem. 3, 885–897. 10.2174/1568026033452249 12678838

[B5] Chang C. L. Liou J. (2016). Homeostatic regulation of the PI(4,5)P2-Ca(2+) signaling system at ER-PM junctions. Biochim. Biophys. Acta 1861, 862–873. 10.1016/j.bbalip.2016.02.015 26924250 PMC4907847

[B6] Conde K. Fang S. Xu Y. (2023). Unraveling the serotonin Saga: from discovery to weight regulation and beyond - a comprehensive scientific review. Cell. Biosci. 13, 143. 10.1186/s13578-023-01091-7 37550777 PMC10408233

[B7] Davey K. J. O'Mahony S. M. Schellekens H. O'Sullivan O. Bienenstock J. Cotter P. D. (2012). Gender-dependent consequences of chronic olanzapine in the rat: effects on body weight, inflammatory, metabolic and microbiota parameters. Psychopharmacol. Berl. 221, 155–169. 10.1007/s00213-011-2555-2 22234378

[B8] EscartíN PéREZ R. E. Mancilla DíAZ J. M. CortéS Salazar F. LóPEZ Alonso V. E. FloráN GarduñO B. (2021). CB1/5-HT/GABA interactions and food intake regulation. Prog. Brain Res. 259, 177–196. 10.1016/bs.pbr.2021.01.005 33541676

[B9] Fernø J. Varela L. Skrede S. VáZQUEZ M. J. Nogueiras R. DiéGUEZ C. (2011). Olanzapine-induced hyperphagia and weight gain associate with orexigenic hypothalamic neuropeptide signaling without concomitant AMPK phosphorylation. PLoS One 6, e20571. 10.1371/journal.pone.0020571 21695181 PMC3113797

[B10] Garro-MartíNEZ E. Fullana M. N. Florensa-Zanuy E. Senserrich J. Paz V. Ruiz-Bronchal E. (2021). mTOR knockdown in the infralimbic Cortex evokes A depressive-like State in mouse. Int. J. Mol. Sci. 22, 8671. 10.3390/ijms22168671 34445375 PMC8395521

[B11] Goit R. K. Taylor A. W. Lo A. C. Y. (2022). The central melanocortin system as a treatment target for obesity and diabetes: a brief overview. Eur. J. Pharmacol. 924, 174956. 10.1016/j.ejphar.2022.174956 35430211

[B12] GóMEZ-Revuelta M. Pelayo-TeráN J. M. Juncal-Ruiz M. VáZQUEZ-Bourgon J. SuáREZ-Pinilla P. Romero-JiméNEZ R. (2020). Antipsychotic treatment effectiveness in first episode of psychosis: PAFIP 3-Year Follow-Up randomized clinical trials comparing Haloperidol, olanzapine, risperidone, aripiprazole, quetiapine, and ziprasidone. Int. J. Neuropsychopharmacol. 23, 217–229. 10.1093/ijnp/pyaa004 31974576 PMC7177160

[B13] Liu X. Lan X. Zhang X. Ye H. Shen L. Hu M. (2024). Olanzapine attenuates 5-HT2cR and GHSR1a interaction to increase orexigenic hypothalamic NPY: implications for neuronal molecular mechanism of metabolic side effects of antipsychotics. Behav. Brain Res. 463, 114885. 10.1016/j.bbr.2024.114885 38296202

[B14] Ma J. Zhang L. Huang Z. Wang G. (2024). Clinical patterns of metabolic syndrome in young, clinically stable, olanzapine-exposed patients with schizophrenia. Ann. Gen. Psychiatry 23, 46. 10.1186/s12991-024-00532-y 39538330 PMC11562516

[B15] Marteene W. Winckel K. Hollingworth S. Kisely S. Gallagher E. Hahn M. (2019). Strategies to counter antipsychotic-associated weight gain in patients with schizophrenia. Expert Opin. Drug Saf. 18, 1149–1160. 10.1080/14740338.2019.1674809 31564170

[B16] Meltzer H. Y. Massey B. W. (2011). The role of serotonin receptors in the action of atypical antipsychotic drugs. Curr. Opin. Pharmacol. 11, 59–67. 10.1016/j.coph.2011.02.007 21420906

[B17] Meng S. Cao H. Huang Y. Shi Z. Li J. Wang Y. (2023). ASK1-K716R reduces neuroinflammation and white matter injury *via* preserving blood-brain barrier integrity after traumatic brain injury. J. Neuroinflammation 20, 244. 10.1186/s12974-023-02923-6 37875988 PMC10594934

[B18] Miyamoto S. Miyake N. Jarskog L. F. Fleischhacker W. W. Lieberman J. A. (2012). Pharmacological treatment of schizophrenia: a critical review of the pharmacology and clinical effects of current and future therapeutic agents. Mol. Psychiatry 17, 1206–1227. 10.1038/mp.2012.47 22584864

[B19] Nectow A. R. Schneeberger M. Zhang H. Field B. C. Renier N. Azevedo E. (2017). Identification of a brainstem circuit controlling feeding. Cell. 170, 429–442.e11. 10.1016/j.cell.2017.06.045 28753423

[B20] Pagi R. (2026). Neuro-metabolic disruption underlying olanzapine-induced obesity. J. Integr. Transl. Biomed. 1, 45–54. 10.67316/jintb.v1.i2.18

[B21] Patel P. Naik M. U. Golla K. Shaik N. F. Naik U. P. (2019). Calcium-induced dissociation of CIB1 from ASK1 regulates agonist-induced activation of the p38 MAPK pathway in platelets. Biochem. J. 476, 2835–2850. 10.1042/BCJ20190410 31530712 PMC7071732

[B22] Peng Y. Feng C. Peng S. Wang Y. Zhang Q. Jiao Z. (2025). GLP-2 prevents antipsychotics-induced metabolic dysfunction in mice. Nat. Metab. 7, 730–741. 10.1038/s42255-025-01252-7 40114026

[B23] Pereira S. Castellani L. N. Kowalchuk C. Alganem K. Zhang X. Ryan W. G. (2024). Olanzapine's effects on hypothalamic transcriptomics and kinase activity. Psychoneuroendocrinology 163, 106987. 10.1016/j.psyneuen.2024.106987 38340539 PMC10947847

[B24] Qi X. Y. Vahdati Hassani F. Hoffmann D. Xiao J. Xiong F. Villeneuve L. R. (2021). Inositol trisphosphate receptors and nuclear calcium in atrial fibrillation. Circ. Res. 128, 619–635. 10.1161/CIRCRESAHA.120.317768 33375812 PMC8243404

[B25] RamíREZ O. A. CóRDOVA A. Cerda M. Lobos P. HäRTEL S. Couve A. (2021). Ryanodine receptor-mediated Ca(2+) release and atlastin-2 GTPase activity contribute to IP(3)-induced dendritic Ca(2+) signals in primary hippocampal neurons. Cell. Calcium 96, 102399. 10.1016/j.ceca.2021.102399 33812310

[B26] Sanz-Castillo B. Hurtado B. Vara-Ciruelos D. EL Bakkali A. Hermida D. Salvador-Barbero B. (2023). The MASTL/PP2A cell cycle kinase-phosphatase module restrains PI3K-Akt activity in an mTORC1-dependent manner. Embo J. 42, e110833. 10.15252/embj.2022110833 36354735 PMC9841333

[B27] Smith M. Hopkins D. Peveler R. C. Holt R. I. Woodward M. Ismail K. (2008). First-v. Second-generation antipsychotics and risk for diabetes in schizophrenia: systematic review and meta-analysis. Br. J. Psychiatry 192, 406–411. 10.1192/bjp.bp.107.037184 18515889

[B28] Song H. J. Jeon I. S. Kim S. R. Park K. S. Soh J. W. Lee K. Y. (2022). PKC-β modulates Ca(2+) mobilization through Stim1 phosphorylation. Genes Genomics 44, 571–582. 10.1007/s13258-022-01230-3 35254656 PMC9042968

[B29] Tao X. Liu H. Xia J. Zeng P. Wang H. Xie Y. (2022). Processed product (Pinelliae Rhizoma Praeparatum) of Pinellia ternata (Thunb.) Breit. Alleviates the allergic airway inflammation of cold phlegm *via* regulation of PKC/EGFR/MAPK/PI3K-AKT signaling pathway. J. Ethnopharmacol. 295, 115449. 10.1016/j.jep.2022.115449 35688257

[B30] Vahid-Ansari F. Albert P. R. (2021). Rewiring of the serotonin system in major depression. Front. Psychiatry 12, 802581. 10.3389/fpsyt.2021.802581 34975594 PMC8716791

[B31] Zhang H. Sun Y. Huang Z. Wu Z. Ying Y. Liu R. (2024). Jiawei-xiaoyao pill elicits a rapid antidepressant effect, dependent on activating CaMKII/mTOR/BDNF signaling pathway in the hippocampus. J. Ethnopharmacol. 318, 117016. 10.1016/j.jep.2023.117016 37567427

